# Tips and tricks for building a good paper: what editors want

**DOI:** 10.1186/s40634-020-00273-3

**Published:** 2020-07-28

**Authors:** Jon Karlsson, Bruce Reider, Edward M. Wojtys, Stefano Zaffagnini

**Affiliations:** 1 Department of Orthopedics, Institute of Clinical Sciences, Sahlgrenska Academy, University of Gothenburg, Gothenburg, Sweden; 2 Department of Orthopaedic Surgery and Rehabilitation, University of Chicago, Chicago, IL U.S.A.; 3 MedSport, Department of Orthopaedic Surgery, University of Michigan, Ann Arbor, MI USA; 4 II Clinica Ortopedica e Traumatologica, IRCCS Istituto Ortopedico Rizzoli, University of Bologna, Via Cesare Pupilli 1, Bologna, BO Italy

## Abstract

ESSKA is constantly committed to promoting the improvement of scientific quality through the publication of books and the organization of dedicated conferences. In line with this commitment, this interview paper was crated with the aim of being useful for all the young scientists and orthopaedics keen in musculoskeletal and sport medicine research. Three Editors from the most important journals in our field were invited to participate: Jon Karlsson from *Knee Surgery Sport Traumatology and Arthroscopy*, Bruce Reider from *The American Journal of Sport Medicine* and Edward Wojtys from *Sports Health*.

## Introduction

The *Journal of Experimental Orthopedics* is a young and relatively small journal, but one that dreams big. In the last months, I am proud to say, the quality of the published articles has been improving. The group of Editors is hard-working and committed to improving the quality of the papers more and more. However, it is not all because of us; the scientific background of each of us was created after years of commitment and publications in important journals; and I myself have been inspired and learnt from the editors of the most prestigious one. As you know, ESSKA is constantly committed to promoting the improvement of scientific quality through the publication of books [1] and the organization of dedicated conferences to which important personalities are invited. In line with this commitment, I decided to create an interview paper that would have been useful both for us and for all the young scientists and doctors keen in musculoskeletal and sport medicine research. Three Editors from the most important journals in our field were invited to participate: Jon Karlsson from *Knee Surgery Sport Traumatology and Arthroscopy*, Bruce Reider from *The American Journal of Sport Medicine* and Edward Wojtys from *Sports Health*. Fourteen questions were created, some dealing with the best way to design and report a scientific paper, some others on the current and future directions of the scientific research. It is for sure an honor to discuss with these Editors and have their opinions and answers since their experience is undoubtful and extremely valuable.

Here below you will find the complete interview. Every question was answered independently by the Editors, to give a broader view of the different perspectives.

## 1-(SZ): The title of a paper is the first thing to read and its value must not be underestimate; which features attract attention of the editor the most? How can a researcher be sure to have an intriguing title?

(JK): The title must be short. It should also “catch the eye” of the reader, but be serious and correct at the same time. I prefer one line only, maximum two lines; never long. And, the title should be a statement related to the main finding in the present study and never a question. In terms of the title, don’t be in doubt, instead be proud of what you found.

(BR): Succinct and descriptive; keep to one line if at all possible. May describe what was done (my personal preference) or the most important result (but do not over-simplify or sensationalize the findings)

(EW): The title of the paper is extremely important. It should draw readers to the paper. No matter how good the research paper is, without an attractive title, many readers will not see it. The title should never be longer than one sentence. Long titles often diffuse the importance of the work.

## 2-(SZ): The abstract is probably the section of the paper that will have the most diffusion, it will be possible to find it on the main databases. It should be a short report of the contents, but what is not to be missed?

(JK): We should remember that the Abstract is sometimes or even often the only part of the article that is read, and this is very much understandable. Many orthopaedic surgeons like to follow what happens in the orthopaedic field in general and therefore they read short-hands or abstracts of many papers that are not their main focus area. Therefore, the abstract should be “short and sharp”. For instance; no introduction and minimal discussion. Conclusion with clinical relevance always needs to be highlighted and may never be missed.

(BR): Include actual results that show the magnitude of the principal outcomes; not just *p* values or general statements.

(EW): The abstract must be a concise report of the research work. Many readers will not go beyond the abstract. They look at the abstract as a short form of the paper. Everything important from the paper must be summarized in the abstract. The background or introduction should be very short. A couple of sentences usually will do. A precise statement about the methods is always needed. Most important is a concise summary of the results. The conclusion should put the work in perspective and entice readers to read the entire work.

## 3-(SZ): What is the importance that you are giving to the study design? In-vitro or in-vivo has the same value in your opinion?

(JK): Correct methods, including a sound and correct description of the study design is the cornerstone of every scientific study. Methods, no matter if they are in-vitro or in-vivo must be reported in such a way that they are understandable and other researchers can – without too much trouble and extra work – repeat them in a second (confirmatory) study.

(BR): The study design should fit the level of existing knowledge in the literature. In vitro is one more step removed from clinical medicine; may be important but relevance to clinical medicine must be clear or it should be in a basic research journal. The same is of course also true for in vivo in animals, but such research is inherently closer to clinical medicine than in vitro.

(EW): As an editor, after I read the abstract, I go directly to the methods of the paper. If the methods are faulty, usually I will not read further. Today’s literature is filled with publications that are faulty in their methods. Being able to focus on the well-done literature is crucial for most readers. The best way to do that is to look critically at the methods. The methods should be in detail enough so that an interested investigator would be able to repeat the study based on the instructions of the methods.

## 4-(SZ): How to build the introduction in a sharp way without being too long? How to raise the interest of the reader for the study without going out of topic?

(JK): Again, this section must be “short and sharp”. Never more than one manuscript page. In fact, the Introduction can be 4 sentences only, i.e. why is the study needed, what is the gap in literature you would like to bridge, and at the end aim/purpose and hypothesis. All too often hypothesis is not mentioned. And, we should remember that the two last items lead up to the statistical analysis, with one (not two or more) primary variables.

(BR): Emphasize how the information yield from the study will be clinically useful. The Need to Know!

(EW): The introduction sets the stage for the research work. It should answer the question- why is this work needed? Good studies identify a knowledge gap that the research paper fills. Most introductions do not require references. If an author starts off with a long review of the literature in the introduction, that counts against the paper. The last sentence or two in the introduction should clearly state the hypothesis which sets the stage for the statistical analysis in the methods.

## 5-(SZ): Material and methods should be like a cooking recipe, but it is not always easy to assemble every concept. What is, in your opinion, the best order to follow to succeed in this section?

(JK): Maybe, it does not matter so much, but I always like to start with the ISB (Ethical) Approval and end with statistics. When it comes to statistics, it is (for clinical studies) always necessary with sample size calculation. All too often this is missed or not properly done. As far as I can see, the type-II statistical error is the most common mistake in clinical studies and it must be avoided at any cost. The best order would be; IRB approval, patients, methods (first surgical methods, rehabilitation and clinical follow-up), measurement methods (including accuracy report) and then statistics. The methods section must be comprehensive and meticulous. This means that the author may need 3–4 pages to describe all methods in detail.

(BR): Chronological.

(EW): The first sentence in the methods for every clinical study should start with the IRB approval of the project. If this statement is not present, the authors should explain why. By far the most common mistake made by authors is the failure to perform a sample size estimate prior to beginning the study. If authors report no significant difference between groups or treatment types without a sample size estimate, I do not read any further. Type 2 statistical errors are extremely common.

A very common mistake, especially in basic science papers, is the failure to report on the reproducibility of the measurement technique. Accuracy of technique and repeatability must be established in each investigation or reference another paper with the exact same technique performed by the same authors. The methods section is the most meticulous portion of a manuscript and should be quite detailed. The last part of the methods is the statistical analysis which should allow clinicians to feel comfortable with the methods.

## 6-(SZ): Of course, clinical and pre-clinical studies have very different designs and aims, but which are the main points to be reported in the methods of these types of study respectively? And in which order?

(JK): For me, working as an Editor for a clinical journal, the basic science studies are very important. They are the foundation that we can build the treatment of patients on. Therefore, the accuracy of methods is most important and for a basic science study, it is important that the clinical relevance is considered.

(BR): Indications for the procedure or inclusion in the study. A clear description of how many patients were eligible for the study and how many actually were studied. How follow-up was conducted and the primary outcome measures.

(EW): As stated in #5, the accuracy of measurement techniques and the reliability and reproducibility must be clarified in the methods and results. If authors report fractions of a degree in measuring range of motion, they must have a technique that allows them to do that. In most cases, that doesn’t exist.

## 7-(SZ): It is true that controlled trials studies are the most appealing, but in the orthopedics field several studies are designed as case series. Therefore, which are the main features that makes a case series interesting for a top-level editor?

(JK): Approximately 50% of studies in our field are case series and maybe 15% level I. Important case series study brings something new, and is not just a repetition of well-known facts. Main points are cohort size, length of follow-up and low dropout rate. Registries are being increasingly used and they are very valuable, but not instead of level I studies. They complement each other.

(BR): Novelty; prospective methods; clearly stated inclusion criteria/indications for the procedure; high rate of follow-up; clinically important outcomes chosen for reporting; transparent description of how the follow-up was done.

(EW): Randomized clinical trials (RCTs) are extremely difficult to perform in Orthopaedics. Also, they usually can answer only one question. Outside of some major medical centers, in many situations, they are not possible. Therefore, we rely on case series that are well-done, hopefully level 1. The most attractive case series have a large cohort, are structured correctly, have a good follow-up rate, and are able to maintain follow-up on at least 80–85% of the patients.

## 8-(SZ): In your opinion which are the statistical mistakes that makes the paper unacceptable?

(JK): As mentioned above, the most common error is the type-II error, which in most cases is due to too limited cohort. We should also remember that sample size calculation is always needed and always necessary in clinical studies. And, we should always avoid to rely too heavily on subgroup analysis. One primary variable is good, not two or three.

(BR): These usually can be corrected, but the paper may be unacceptable if the corrections will lead to a different result; Also repeatedly citing “trends” or describing non-significant differences as “differences”.

(EW): The absence of a sample size estimate in a clinical study is the most common statistical mistake that I see. It’s unfortunate, because most research manuals stress the importance of power analysis and sample size estimate repeatedly. Yet, in many papers, it isn’t there.

## 9-(SZ): How do you want the results to be presented? Some authors prefer to describe them, while others prefer several tables and figures; which are the pros and cons and what to avoid?

(JK): Again, short is good. My advice is always” *use tables and figures for details and avoid long text”.* This means that you report on the important findings in words and the rest in tables. In general, the Results section should be no more than one manuscript page (this is, of course, not written in stone, however).

(BR): Very complete tables with the most important results pointed out in the text.

(EW): In the results section, I like to see all data in tabular form. Whenever this is possible, that is the preferred technique. Minimizing text in the results section is best. Only use the text for those portions of the results that cannot be put in tables or figures.

## 10-(SZ): It is easy of lost focus in the discussion section, making it too long and not interesting. What is the editor looking for in the discussion? What to avoid?

(JK): A good rule is” *you don’t need to tell the readers everything you know”*. I look for four (4) parts in the Discussion section. To start with, the main finding(s) of your study. Then, the researchers need to put their work in context, what is already know and do you confirm or discard. If your new findings are really new, you need to explain well and build a case, often using multiple references (remember that references should be as recent as possible). Then I look for a sound and truthful description of limitations and finally a sentence about clinical usefulness. Limitations are always necessary; authors should never try to hide limitations in their paper. Limitations can lead to new and important studies. Also, don’t write what is already very well know, like “Hip fractures are very common in elderly women”. The paper will not be better for writing like this, just longer and probably boring.

(BR): Most important findings. Clinical relevance of these findings. How and why these findings agree or disagree with existing literature. Limitations of the study honestly described. Factual conclusions.

(EW): The most common mistake that I see in the discussion section is a repetition of the results. That frequently increases the length of the discussion and the paper unnecessarily. I look for a clinic scientific evaluation of the work in the discussion with relevant references only. Too often, inexperienced writers will include every reference on the topic. Many of those references are no longer relevant. The last portion of this discussion is extremely important. It is the limitation section. If an author truly recognizes the limitations of their work and can put it into proper perspective, that goes a long way in my evaluation of a paper. Most revisions of papers would do wise to include critical comments from the reviewers in their limitations section. These are usually quite helpful and identify the weaknesses and strengths of a paper.

## 11-(SZ): It is possible to change the Editor’s mind and decision about a paper, with a well-constructed discussion? If yes, where is the secret?

(JK): Yes, absolutely. The secret (this is no secret really) is to be polite and communicate in constructive spirit. We always take complaints seriously and what we do in practical terms is to move the manuscript to another Editor, who will start fresh, maybe his/her own (new) review or maybe select new reviewers. And then we communicate in the same manner. This is very important. Mutual respect is important.

(BR): Maybe not, but see answer to #10 above. Ned to explain why the study is valuable despite its limitations.

(EW): Editors are human beings and they do make mistakes. If someone is not happy with a decision that I’ve rendered about their paper, I will listen to their rebuttal. If the reviews are done well, the likelihood of a change in decision is small, but it does happen.

## 12-(SZ): Authors must be honest about the limitation of their study, what number and what type of limitation makes the paper unacceptable?

(JK): There is no absolute rule; honesty is most important.

(BR): No set number of limitations. Every study has limitations. It’s often a judgment call that may vary depending upon the uniqueness of the subject and the quality of studies already in the literature. If the limitations cause the results to be unreliable (e.g. large loss to follow-up, biased assessment of results) that can make the paper unacceptable.

(EW): There is no exact number that makes for too many limitations. Quite often the best papers correctly identify their limitations and many times they are extensive. I think this does help the reader put the paper’s results in perspective.

## 13-(SZ): The scenario is dramatically changing in sport medicine research; how do you think it will evolve? Which kind of papers will you accept in 5 to 10 years? More long term data, innovative technologies, regenerative medicine, what else?

(JK): This is difficult; probably all of this. I would guess that regenerative medicine will play a major role in future. Here, we need to bridge the gap between basic science and clinical outcome studies. Registries will also enable us to rely more and more on “big data”.

(BR): Yes to the above. More controlled studies. Subjective AND objective follow-up of clinical studies.

(EW): The field of sports medicine is dramatically changing and in years to come we will see more and more regenerative medicine, long-term data from registries, and hopefully innovative technologies. As editors, the challenge will be to keep up in these fields and to continue to identify capable associate editors and reviewers to properly review the submitted papers. For me, this is a constant challenge. It is a welcome one because it has kept me close to the cutting edge but does present challenges.

## 14-(SZ): Over the past few years we have seen an increasing number of submissions from non-western research groups; what do you think are the greatest potentials of these groups? How to balance a journal’s publications, to obtain a well-rounded vision of the scientific scenario?

(JK): I have during my time as Editor seen the rise of Japanese and Korean researchers, and lately studies from China are increasing both in quantity and quality. This is good. But, still we see too few studies from South-America and East Europe. We should work together and for instance we could give web-based courses on study design and manuscript writing in these parts of the world.

(BR): Accept the best studies, wherever they originate.

(EW): *Sports Health* is a relatively new journal. Initially, most of our papers came from North America. However, our submissions now include South America, Europe, Australia, Africa, China, and Japan. I think this diversity reflects well on Sports Medicine research and will be a strength in the future. The challenge will be to continue to incorporate all of the different views and approaches of different researchers to find what’s best for our patients.

## Conclusions

This interesting collection of answers has given us different points of view on sport medicine research. Among the interview questions, I tried to raise the most challenging and frequent issues I have met during my scientific experience, and, despite I am actually editor in chief of an ESSKA journal, I have found extremely useful all the opinions provided by such leading and experts scientific editors. Most of the issues covered in the editors’ answers represent a useful tool not only for the orthopedics and sports medicine investigators, but also for all the young scientists engaged in different research fields. I think it is not easy for a young researcher to put together all the numerous pieces that make up a good paper and I hope that this interview may have clarified the ideas and given some tricks. I am sure that in the next article you write, you will keep these valuable tips in mind that will improve their quality. Finally, a big thanks to these three friends and colleagues that have been available to spend time and effort in providing the responses for this article that every researcher, I think, should have on his or her desk.

## References

[1] Musahl V, Karlsson J, Hirschmann MT, Ayeni OR, Marx RG, Koh JL, Nakamura N, Musahl V, Karlsson J, Hirschmann MT, Ayeni OR, Marx RG, Koh JL, Nakamura N (2019). Basic Methods Handbook for Clinical Orthopaedic Research.

